# Early Depression Screening Is Feasible in Hospitalized Stroke Patients

**DOI:** 10.1371/journal.pone.0128246

**Published:** 2015-06-03

**Authors:** Rahul R. Karamchandani, Farhaan Vahidy, Suhas Bajgur, Kim Yen Thi Vu, H. Alex Choi, Robert Kirk Hamilton, Mohammad H. Rahbar, Sean I. Savitz

**Affiliations:** 1 Department of Neurology, Stroke Program, University of Texas-Houston Medical School, Houston, TX, United States of America; 2 Memorial Hermann Hospital, Texas Medical Center, Houston, TX, United States of America; 3 Department of Neurology, University of Texas-Houston Medical School, Houston, TX, United States of America; 4 Department of Neurosurgery, University of Texas-Houston Medical School, Houston, TX, United States of America; 5 Division of Clinical and Translational Sciences, Department of Internal Medicine, University of Texas-Houston Medical School, Houston, TX, United States of America; 6 Division of Epidemiology, Human Genetics, and Environmental Sciences, the University of Texas School of Public Health at Houston, Houston, TX, United States of America; University of Münster, GERMANY

## Abstract

**Background and Purpose:**

Post-stroke depression (PSD) is common but is not routinely assessed for in hospitalized patients. As a Comprehensive Stroke Center, we screen all stroke inpatients for depression, though the feasibility of early screening has not been established. We assessed the hypothesis that early depression screening in stroke patients is feasible. We also explored patient level factors associated with being screened for PSD and the presence of early PSD.

**Methods:**

The medical records of all patients admitted with ischemic stroke (IS) or intracerebral hemorrhage (ICH) between 01/02/13 and 15/04/13 were reviewed. A depression screen, modified from the Patient Health Questionnaire-9, was administered (maximum score 27, higher scores indicating worse depression). Patients were eligible if they did not have a medical condition precluding screening. Feasibility was defined as screening 75% of all eligible patients.

**Results:**

Of 303 IS and ICH inpatients, 70% (211) were eligible for screening, and 75% (158) of all eligible patients were screened. More than one-third of all patients screened positive for depression (score > 4). Women (OR 2.06, 95% CI 1.06–4.01) and younger patients (OR 0.97, 95% CI 0.96–0.99) were more likely to screen positive. Screening positive was not associated with poor discharge/day 7 outcome (mRS > 3; OR 1.45, 95% CI 0.74–2.83).

**Conclusions:**

Screening stroke inpatients for depression is feasible and early depression after stroke is common. Women and younger patients are more likely to experience early PSD. Our results provide preliminary evidence supporting continued screening for depression in hospitalized stroke patients.

## Introduction

Depression after stroke is prevalent in approximately one-third of survivors[1]. Prior studies have demonstrated an association between post-stroke depression (PSD) and physical functional impairment[2, 3], including the severity of impairment in activities of daily living[4]. In addition, PSD has been linked to increased risk of recurrent stroke[5], poor long-term functional outcome[6], and increased risk of mortality after stroke[7, 8].

The importance of detecting depression in stroke patients is now emphasized as a requirement for Joint Commission-sponsored Comprehensive Stroke Center (CSC) certification[9]. Despite the CSC requirement, there is no literature describing how to best assess depression in the acute post-stroke period. It is unknown if depression can be detected during this time frame and how screening can potentially affect clinical care.

We sought to determine the feasibility of screening hospitalized stroke patients for depression and examined patient level factors associated with being screened for PSD. Also, we explored factors associated with early PSD as well as the relationship between early depression after stroke and short-term functional outcome. We hypothesized that early screening for depression in hospitalized stroke patients is feasible.

## Methods

### Ethics Statement and Setting

Medical records of all patients admitted to Memorial Hermann Hospital-Texas Medical Center (Houston, TX) with a clinical diagnosis of ischemic stroke (IS) or intracerebral hemorrhage (ICH) between 01/02/13 and 15/04/13 were reviewed with permission from the Institutional Review Boards of Memorial Hermann Hospital and The University of Texas Medical School-Houston. All patient data was de-identified and analyzed anonymously. The study was retrospective, and post-hoc consent was not obtained due to the logistical difficulties that this presented.

**Fig 1:**
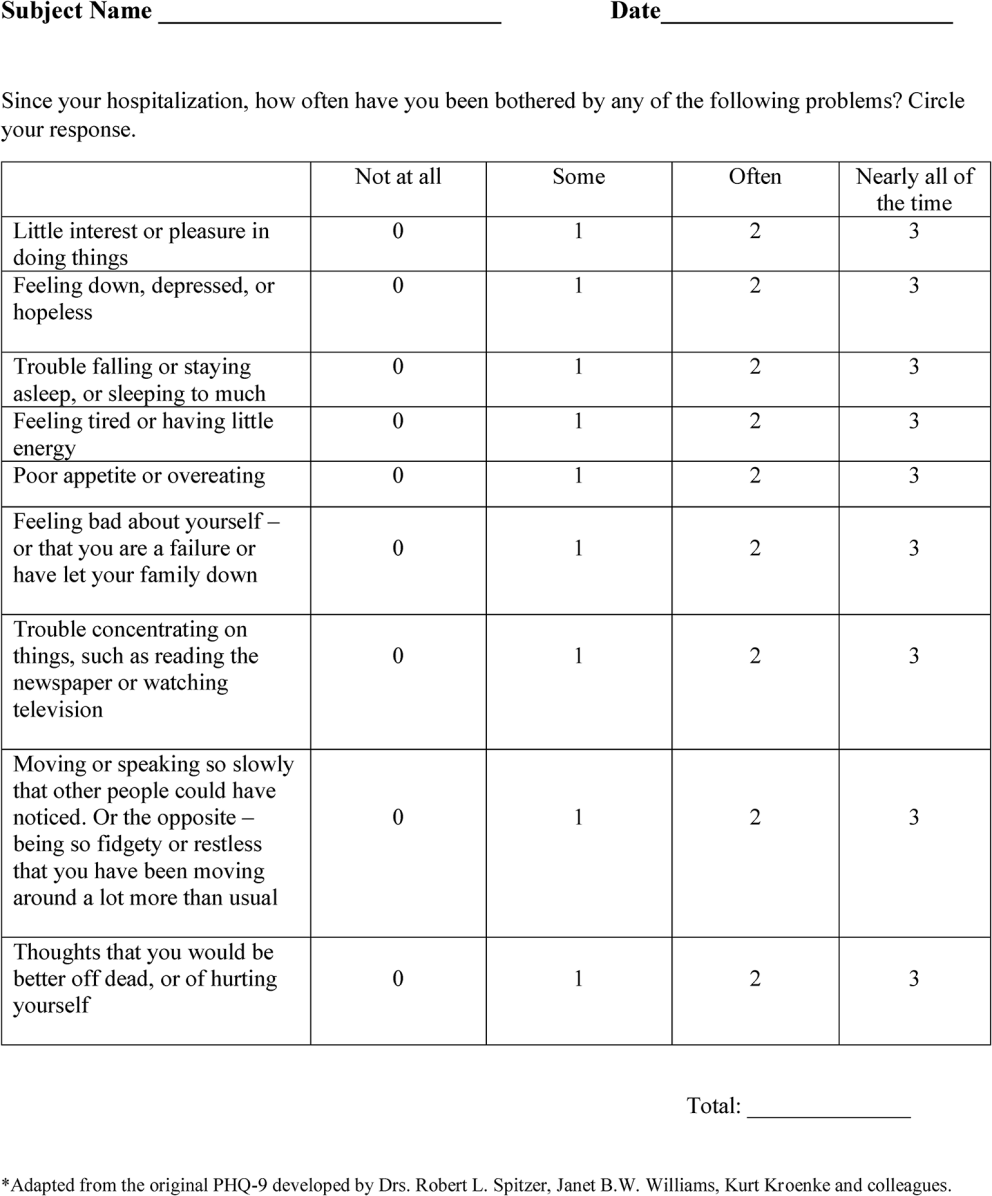
Modified Patient Health Questionnaire–9 (PHQ-9). PHQ-9 Scores and Depression Severity**. PHQ-9 Score: 0–4: none-minimal; 5–9: mild; 10–14: moderate; 15–19: moderately severe; 20–27: severe. **Kroenke K, Spitzer RL. Psychiatric Annals 2002;32:509–521.

Beginning 01/02/13, a depression screen, modified from the Patient Health Questionnaire (PHQ-9)[10], was administered to all IS and ICH inpatients as part of the screening requirement for a Joint Commission CSC (Fig 1). Demographic variables, patient co-morbidities, stroke characteristics, and outcome data were collected prospectively as part of the University of Texas Houston Stroke Registry[11].

### Eligibility and Participants

Eligible patients were defined as those who did not have a medical condition precluding screening, such as those who were made comfort care, placed into hospice, or died during the acute hospitalization. Patients were also deemed ineligible if they had language dysfunction. While tools exist to assess depression in aphasic patients[12–14], no depression screening instrument has been found to be definitively suited for this patient population[15]. If a patient was unable to be screened during the acute period of hospitalization due to a medical or neurologic condition (i.e. depressed level of consciousness due to cerebral edema or encephalopathy due to co-incident infection), the screen was attempted again at a later time. All attempts were made to screen patients prior to hospital discharge or transfer to another service.

### Screening Instrument and Variables

**Fig 2:**
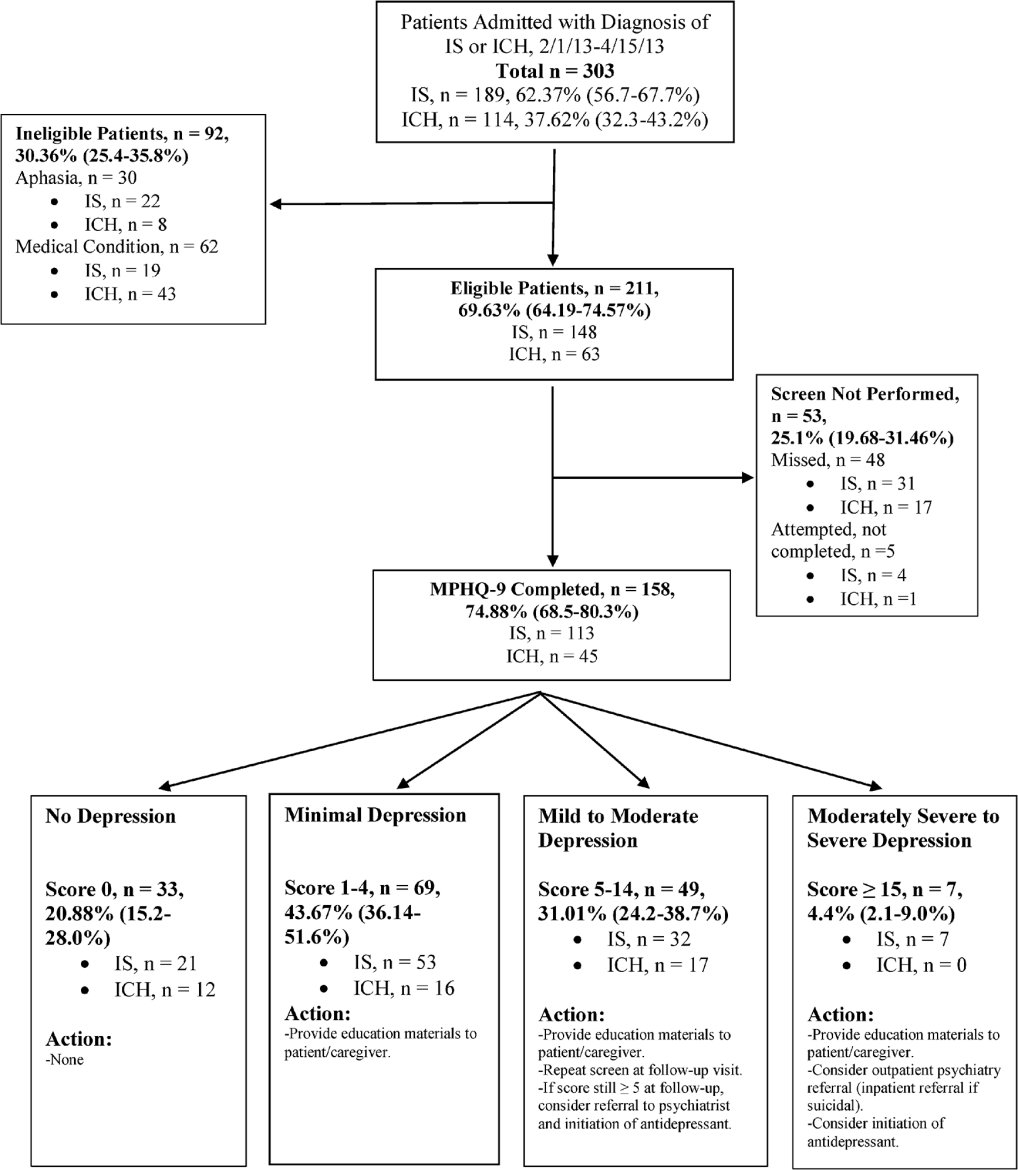
Study Flow-Diagram and Summary of Depression Scores. IS = ischemic stroke. ICH = intracerebral hemorrhage.

The PHQ-9 has been studied in stroke patients[16, 17] and was modified from its original version in order to detect depressive symptoms since the time of hospitalization, rather than in the preceding two weeks. Scores range from 0 (no depression) to 27 (severe depression)[10]. The modified PHQ-9 (MPHQ-9) was self-administered, as is the original, and scores were collected and recorded by social workers. Social workers recorded the score in the electronic medical record and communicated with the primary medical team if a patient scored > 4 (mild to moderate depression). The corresponding action plan was then carried out by the social worker and medical team (Fig 2). Screens that were not completed were categorized as being not attempted due to aphasia; not completed due to a medical condition (death, hospice/comfort measures, prolonged intubation); missed (screen not ordered, or ordered and not completed); or attempted and not completed (patient refusal or patient not available at time despite multiple social work visits).

Patients with at least minimal depression (score ≥ 1) were provided with a packet of education materials consisting of a PSD pamphlet and fact-sheet, as well as information on other post-stroke mood disorders. Phone numbers for local counseling services were also provided, as well as informational website addresses for local and national mental health organizations. Our complete institutional algorithm for depression screening is shown in Fig 2.

### Study Objectives and Timeline

The primary objective of the study was to investigate the feasibility of screening for PSD by evaluating the percentage of patients screened in each group. Feasibility was defined as screening at least 75% of all eligible IS and ICH patients. We also investigated patient level factors associated with being screened for and screening positive for PSD. Positive screening was defined as a MPHQ-9 score > 4. Prior studies have shown that scores ≥ 10 on the PHQ-9 have moderate sensitivity and specificity for post-stroke depression[16, 17]. A more broad definition was used in our study to cast a wider net in detecting depressive symptoms. Lastly, we examined the relationship between early PSD and poor discharge/day 7 outcome, which was defined as modified Rankin scale (mRS) score of > 3, as in other stroke trials[18].

The 2.5 month period was selected for study in order to evaluate our data collection process, the screening instrument, and our results. As this is a novel area of study, we felt that a thorough process review was necessary after a short initial screening window to assure that the screens were being completed, that our algorithm was being followed, and that appropriate, systems-targeted efforts to improve the process were put into place earlier rather than later.

### Statistical Analysis

Descriptive statistics for all patients, as well as stratified per stroke type, are presented as frequencies for nominal and categorical data, and as means ± standard deviation or median with interquartile range for continuous variables. Proportions with 95% confidence intervals have been reported for feasibility metrics. The MPHQ-9 scale, NIHSS, and mRS were dichotomized for certain analyses (MPHQ-9 > 4 as indication of depression, NIHSS > 15 as moderate to severe stroke, and mRS 4–6 as poor outcome). Univariable and multivariable logistic regression were fit to determine factors associated with MPHQ-9 screening, and for screening positive with the MPHQ-9. Variables were selected for the primary main effects model based on their statistical (p < 0.2) and clinical significance. The final model was checked for goodness of fit. Linear regression models were fit to explore association of continuous MPHQ-9 score with demographic and clinical variables. In both logistic and linear models, important interactions were also assessed. All statistical tests were performed at 5% level of significance using STATA version 12 (STATA Corp. College Station, TX).

## Results

### Baseline Characteristics and Functional Outcomes

Between 01/02/13 and 15/04/13, 303 patients were admitted to the Stroke Service with a clinical diagnosis of acute IS or ICH. Table 1 displays demographics, co-morbidities, and stroke characteristics for each group.

**Table 1:** Patient Demographics, Co-morbidities, and Stroke Characteristics.

	Total Patients	Ischemic Stroke	Intracerebral Hemorrhage
	N = 303	n = 189	n = 114
Age, mean(y) ± SD	63.82 ± 15.94	64.89 ± 16.48	62.05 ± 14.90
Females, n (%)	135 (44.55)	87 (46.03)	48 (42.11)
Race, n (%)			
White (non-Hispanic)	145 (47.85)	92 (48.68)	53 (46.49)
White (Hispanic)	33 (10.89)	17 (8.99)	16 (14.04)
Black	91 (30.03)	60 (31.75)	31 (27.19)
Asian	5 (1.65)	2 (1.06)	3 (2.63)
Other	16 (5.28)	8 (4.23)	5 (4.39)
NIHSS on arrival, median (IQR)	9 (3 – 20)	7 (3 – 18)	16 (6 – 27.5)
Stroke Etiology (IS), n (%)			
Cardioembolic		61(32.28)	-
Large Vessel		24 (12.70)	-
Small Vessel		26 (13.76)	-
Unknown		54 (28.57)	-
Other		17 (8.99)	-
Stroke Etiology (ICH), n (%)			
Hypertension		-	64 (56.64)
Amyloid		-	5 (4.42)
Coagulopathy		-	11 (9.73)
Aneurysm		-	1 (0.88)
AVM		-	3 (2.65)
Neoplasm		-	2 (1.77)
Unknown		-	19 (16.81)
Other		-	6 (5.31)
Multiple		-	2 (1.77)
Smoker, n (%)	70 (23.10)	50 (24.46)	20 (17.54)
Atrial Fibrillation, n (%)	64 (21.12)	52 (27.51)	12 (10.53)
IV tPA therapy, n (%)		62 (32.8)	-
Intra-arterial therapy, n (%)		7 (3.7)	-
Glucose, mean(mg/dL) ± SD	142.38 ± 62.63	134.08 ± 64.19	156.41 ± 57.5
Hemicraniectomy, n (%)	17 (5.6)	4 (2.12)	13 (11.4)
Hypertension, n (%)	224 (73.93)	140 (74.07)	84 (73.68)
Hyperlipidemia, n (%)	101 (33.33)	74 (39.15)	27 (23.68)
Diabetes, n (%)	100 (33.0)	68 (35.98)	32 (28.07)

Ischemic stroke and ICH groups were not significantly different with respect to age, gender, and race. Intracerebral hemorrhage patients had higher median NIHSS scores on admission compared to IS patients. Nearly 33% of all IS patients admitted were treated with intravenous alteplase, consistent with prior data published from our institutional registry[11]. A significantly higher (p<0.001) proportion of ICH patients had poor outcome, as compared to IS patients (Table 2).

**Table 2:** Discharge Disposition and Functional Outcomes.

Discharge disposition, n (%)	Total Patients (n = 294)	Ischemic Stroke (n = 182)	Intracerebral Hemorrhage (n = 112)
Home	88 (29.04)	66 (36.26)	22 (19.64)
In-patient Rehabilitation	23 (7.59)	14 (7.69)	9 (8.04)
SNF/Sub-acute/LTAC	10 (3.30)	6 (3.30)	4 (3.57)
Transfer other service	121 (39.93)	79 (43.41)	42 (37.50)
Hospice/Death	47 (15.51)	14 (7.69)	33 (29.46)
Other	5 (1.65)	3 (1.65)	2 (1.79)
mRS Discharge, median (IQR)	4 (3–5)	3.5 (2–4)	5 (4–5)
mRS Discharge (4–6), n (%)	178 (58.75)	91 (48.15)	87 (76.32)

SNF = skilled nursing facility, LTAC = long-term acute care facility, mRS = modified Rankin scale score.

### Feasibility of Screening for Post-Stroke Depression

Of the 303 total IS and ICH patients, 211 (70%) were eligible for screening, and 75% (158) of eligible patients were screened (Fig 2). Eligible patients were not screened because they were either missed (n = 48; 23%) or because the screen was not completed, despite being attempted (n = 5; 2%). Of the 92 patients who were ineligible for screening, 30 were aphasic and 62 had a medical condition precluding screening (Fig 2). The median time that the screens were performed for IS and ICH patients were 2 and 3 days after admission, respectively. For the entire cohort, the median time for screening was 2.5 days after admission.

### Factors Associated with Being Screened for Post-Stroke Depression

The likelihood of being screened, if eligible, was not found to be associated with any demographic or clinical factors (Table 3).

**Table 3:** Factors Associated With Being Screened for Post-Stroke Depression.

	MPHQ-9 Screened (n = 158)	MPHQ-9 Not Screened (n = 53)	OR (95% CI)	P value
Age, n (%)	62.0 (16.2)	62.9 (14.5)	0.99 (0.97 – 1.01)	0.73
Females, n (%)	72 (45.6)	23 (43.4)	1.09 (0.58 – 2.04)	0.78
Race, n (%)				
White (non-Hispanic)	69 (45.4)	29 (56.9)	Referent Category	
White (Hispanic)	18 (11.8)	3 (5.9)	2.5 (0.69 – 9.22)	0.16
Black	54 (35.5)	17 (33.3)	1.3 (0.66 – 2.67)	0.42
Asian	1 (0.7)	0 (0.0)	-	-
Other	10 (6.6)	2 (3.9)	2.1 (0.43 – 10.19)	0.36
NIHSS on Arrival*	5 (2 – 10)	6 (2 – 19)	1 (-1 – 5)	0.21
Smoker, n (%)	44 (28.9)	11 (22.5)	1.4 (0.66 – 3.0)	0.37
Atrial Fibrillation, n (%)	25 (16.5)	12 (24.0)	0.62 (0.28 – 1.35)	0.23
Glucose, mean(mg/dL) ± SD	135.45 (59.7)	137.5 (62.1)	0.99 (0.99 – 1.00)	0.83
Hypertension, n (%)	114 (72.2)	41 (77.4)	0.76 (0.36 – 1.57)	0.46
Hyperlipidemia, n (%)	54 (34.2)	18 (33.9)	1.01 (0.52 – 1.94)	0.97
Diabetes, n (%)	52 (32.9)	17 (32.08)	1.03 (0.53 – 2.02)	0.91
Ischemic Stroke, n (%)	113 (71.5)	35 (66.0)	0.77 (0.39 – 1.5)	0.45

* *Medians tested for equality using Wilcoxon rank-sum test*, *and 95% CI for difference between medians is reported*

MPHQ-9 = Modified Patient Health Questionnaire-9.

### Depression Scores and Factors Associated with Early Post-Stroke Depression

Median score on the MPHQ-9 for both IS and ICH patients was 3. Overall, more than one-third of all IS and ICH patients screened positive for depression, as defined in our study as a score ≥4. (Fig 2). Nineteen patients out of 158 (12.03%; 95% CI 7.76%-18.16%) scored ≥ 10. Seven patients scored ≥15, all of whom were admitted with IS, and of which 3 had prior depressed mood in the past. One reported benefit from discussing low mood and stressors with a social worker during the hospitalization, and was prescribed an antidepressant. The patient reported a significant improvement in mood with antidepressant treatment at follow-up, and received additional outpatient counseling. Another patient exhibited suicidal thoughts. Inpatient psychiatry consultation was obtained, the patient was initiated on an antidepressant, and outpatient mental health clinic follow-up was arranged. A third was offered outpatient psychiatry referral during stroke clinic follow-up visit due to persistent depressed mood, though the patient declined.

In both univariable (Table 4) and multivariable models, younger age (OR 0.97, 95%CI 0.96–0.99) and female sex (OR 2.06, 95% CI 1.06–4.01) were associated with screening positive for PSD (Table 4). When depression score was treated as a continuous variable, female sex was the only factor associated with a higher score (p = 0.017).

**Table 4:** Factors Associated With Screening Positive for Post-Stroke Depression.

	MPHQ-9 Positive (> 4), (n = 56)	MPHQ-9 Negative (< 4), (n = 102)	OR (95% CI)	P value
Age, mean(y) ± SD	58.4 ± 16.9	64.0 ± 15.6	0.97 (0.96 – 0.99)	0.04
Females, n (%)	32 (57.1)	40 (39.2)	2.06 (1.06 – 4.01)	0.03
Race, n (%)				
White (non-Hispanic)	25 (44.6)	44 (43.1)	Referent Category	
White (Hispanic)	8 (14.3)	10 (9.8)	1.4 (0.49 – 4.03)	0.52
Black	18 (32.1)	36 (35.3)	0.88 (0.42 – 1.86)	0.74
Asian	0.0 (0)	1 (0.9)	-	-
Other	2 (3.6)	8 (7.8)	0.44 (0.08 – 2.23)	0.32
NIHSS on arrival, median (IQR)	6 (3 – 12)	5 (2 – 9)	1.02 (0.97 – 1.06)	0.52
NIHSS > 15, n (%)	10 (17.9)	14 (13.7)	1.33 (0.55 – 3.24)	0.53
Smoker, n (%)	16 (28.6)	28 (27.5)	1.05 (0.51 – 2.18)	0.89
Atrial Fibrillation, n (%)	10 (17.9)	15 (14.7)	1.26 (0.52 – 3.03)	0.61
Glucose, mean(mg/dL) ± SD	131.5 ± 56.8	137.6 ± 61.5	0.99 (0.99 – 1.00)	0.54
Hypertension, n (%)	43 (76.8)	71 (69.6)	1.44 (0.68 – 3.05)	0.38
Hyperlipidemia, n (%)	22 (39.3)	32 (31.4)	1.42 (0.72 – 2.79)	0.32
Diabetes Mellitus, n (%)	18 (32.1)	34 (33.3)	0.95 (0.47 – 1.89)	0.88
Ischemic Stroke, n (%)	39 (69.6)	74 (72.6)	1.15 (0.56 – 2.36)	0.69

MPHQ-9 = Modified Patient Health Questionnaire-9.

### Association with Poor Functional Outcome

Of the 56 patients who screened positive for PSD, outcome data were available for 54. Twenty-seven of these 54 (50%) had a poor outcome (mRS > 3). Of the 98 patients who did not screen positive and had outcome data documented, 40 (41%) had a poor outcome. Though the odds of having a poor outcome with PSD were higher, this difference did not reach the threshold of statistical significance (p = 0.276; OR 1.45, 95% CI 0.74–2.83).

## Discussion

### Key Results and Interpretation

We describe a new clinical practice to screen for depression in the early stages of the hospitalized patient with IS or ICH. During this early phase of implementation of Joint Commission CSC requirements, we were able to successfully screen nearly 75% of all eligible patients, hence reaching our predefined benchmark.

Our screening instrument was novel and was adapted from the original PHQ-9[10]. The original screen detects depressive symptoms within the preceding two weeks, whereas our modified version inquires about symptoms since the time of hospitalization. Our selection of a screening instrument was based on prior data supporting use of the PHQ-9 in subacute stroke patients[16, 17].

The importance of early PSD is highlighted as a CSC requirement, though some may argue that early depression simply reflects a reaction to the acute illness and new disability, rather than persistent, true depression[19]. Prior data have demonstrated that the symptom profile of those with PSD differs from those with major depressive disorder without clinical comorbidity[20]. Also, depressive symptoms at the time of inpatient rehabilitation admission have been shown to correlate with less efficient use of rehabilitative services in stroke patients, underscoring the importance of early diagnosis of PSD[21]. For the seven patients who scored ≥15 in our study, the identification of early depression offered opportunities for further questioning and exploration of symptoms, as well as intervention.

Using the definition of a positive score as > 4 on the MPHQ-9, the overall rate of depression in our subset of IS and ICH patients was approximately 35%, comparable to previously published data for depression in the stroke population as a whole[1]. Our definition was more lenient than in other studies[16, 17]. Nineteen patients (12%) scored ≥ 10. We arbitrarily chose a more broad definition and casted a wider net, as the importance of acute depressive symptoms is a novel area of study, and we did not want to exclude patients from receiving medical attention and resources who may benefit from such interventions. Additional work is necessary to define and validate a score cutoff for detecting depression using the MPHQ-9 in hospitalized stroke patients.

In our study, females and younger patients were more likely to screen positive for depression. In U.S. adults, females have higher depression prevalence rates compared to males[22]. It is possible that an underlying, sex-specific predisposition to depression in females persists in the stroke population as well. The mean age of all patients with a positive depression screen was 58 years compared to 64 years in those screening negative. Depression prevalence rates in the U.S. are higher in younger age groups compared to older (ages 18–25 and 26–49, versus ≥ 50)[22], and our findings may reflect a particular predisposition to depression in a slightly younger age group of the patients studied. Nonetheless, this is also an area that needs further study in the stroke population, specifically.

### Limitations

Pre-morbid depression status and antidepressant use on admission were not routinely collected, and it is likely that some of our patients had depressive symptoms prior to their stroke. Our depression screen was slightly modified from the original PHQ-9, and has not been previously validated in the stroke population. Our score cutoff to define depression was arbitrary, though we also explored factors associated with a higher depression score when the latter was treated as a continuous variable. The timing of screening for depression was not standardized, and this could have had an effect on depression scores. We examined outcome data which was collected at discharge or hospital day 7, as 90-day mRS scores were not consistently collected in our registry for the time period studied. Lastly, more than 1/4 of eligible patients were not screened due to logistical difficulties, though we expect the number of screens “missed” to reduce with targeted systems-improvement efforts.

### Generalizability and Future Directions

As we examined data from the time that our institution began screening on 01/02/13, we anticipate that our processes will continue to evolve and improve in order to screen more patients. A number of these improvements have already taken place, and some of these may be able to be applied at other centers. Electronic medical record order-sets at our center have been modified in order to include a pre-checked depression screen for each patient admitted with IS or ICH. Medical teams and social workers have been educated to be aware of patients admitted on a Friday that may be discharged over the weekend, when social work staff is limited and screens may be missed. Patients that are intubated for multiple days are being re-evaluated by social workers after extubation serially to determine if the screen can be completed. A depression screen template has been created to populate into the electronic record, allowing for automatic tabulation of total scores and facilitating data entry and abstraction. Social workers have been educated to document specific reasons that a screen cannot be completed, as previously this data had to be manually abstracted and was not standardized. Lastly, each patient who is discharged is reviewed with a checklist to assure that all the necessary components have been completed, including a depression screen.

While depression detected 3 months after stroke has been independently associated with poor functional outcome, additional prospective studies are necessary to better understand the relationship between early PSD and patient outcome. If PSD is able to be identified early, this offers the opportunity to institute therapies that may positively impact patient outcomes. This study represents the first step in identifying early PSD by examining the feasibility of screening hospitalized patients for depression during the initial inpatient stay.

## Conclusions

Depressive symptoms after stroke are common and have been linked to poor neurological outcomes and increased mortality risk. Early identification of PSD may offer opportunities for prompt initiation of therapies and prevention of poor outcomes and early death. Our study demonstrates that early screening for depression after stroke is feasible and that specific patient subgroups are more likely to experience PSD. Several system-wide improvements have been put into place at our institution since beginning to screen for PSD in hospitalized stroke patients, and some of these may be able to be applied at other centers. Further studies may focus on additional system improvement efforts to screen for PSD, defining validated criteria to diagnose PSD in hospitalized patients, and examine the significance of early PSD on long-term neurological outcomes, quality of life, and mortality.

## References

[1] Hackett ML, Yapa C, Parag V, Anderson CS. Frequency of depression after stroke: a systematic review of observational studies. Stroke. 2005;36: 1330–40. 15879342 10.1161/01.STR.0000165928.19135.35

[2] van de Weg FB, Kuik DJ, Lankhorst GJ. Post-stroke depression and functional outcome: a cohort study investigating the influence of depression on functional recovery from stroke. Clin Rehabil. 1999;13: 268–72. 10392654 10.1191/026921599672495022

[3] Kauhanen M, Korpelainen JT, Hiltunen P, Brusin E, Mononen H, Määttä R, et al Poststroke depression correlates with cognitive impairment and neurological deficits. Stroke. 1999;30: 1875–80. 10471439 10.1161/01.str.30.9.1875

[4] Sinyor D, Amato P, Kaloupek DG, Becker R, Goldenberg M, Coopersmith H. Post-stroke depression: relationships to functional impairment, coping strategies, and rehabilitation outcome. Stroke. 1986;17: 1102–7. 3810708 10.1161/01.str.17.6.1102

[5] Sibolt G, Curtze S, Melkas S, Pohjasvaara T, Kaste M, Karhunen PJ, et al Post-stroke depression and depression-executive dysfunction syndrome are associated with recurrence of ischaemic stroke. Cerebrovasc Dis. 2013;36: 336–43. 10.1159/000355145 24193249

[6] Pohjasvaara T, Vataja R, Leppӓvuori A, Kaste M, Erkinjuntti T. Depression is an independent predictor of poor long-term functional outcome post-stroke. Eur J Neurol. 2001;8: 315–9. 11422427 10.1046/j.1468-1331.2001.00182.x

[7] Morris PL, Robinson RG, Samuels J. Depression, introversion and mortality following stroke. Aust N Z J Psychiatry. 1993;27: 443–9. 8250788 10.3109/00048679309075801

[8] Bartoli F, Lillia N, Lax A, Crocamo C, Mantero V, Carrá G, et al Depression after stroke and risk of mortality: a systematic review and meta-analysis. Stroke Res Treat. 2013;2013: 862978 10.1155/2013/862978 23533964 PMC3606772

[9] The Joint Commission, All Rights Reserved. Advanced Disease-Specific Care Certification Requirements for Comprehensive Stroke Center (CSC). Effective July 1, 2013. Available: http://www.jointcommission.org/assets/1/18/DSC_CSC_Chap.pdf. Accessed 4 April 2014.

[10] Kroenke K, Spitzer RL, Williams JB. The PHQ-9: validity of a brief depression severity measure. J Gen Intern Med. 2001;16: 606–613. 11556941 10.1046/j.1525-1497.2001.016009606.x PMC1495268

[11] Rahbar MH, Gonzales NR, Ardjomand-Hessabi M, Tahanan A, Sline MR, Peng H, et al The University of Texas Houston Stroke Registry (UTHSR): implementation of enhanced data quality assurance procedures improves data quality. BMC Neurol. 2013;13:61 10.1186/1471-2377-13-61 23767957 PMC3687564

[12] Sutcliffe LM, Lincoln NB. The assessment of depression in aphasic stroke patients: the development of the Stroke Aphasic Depression Questionnaire. Clin Rehabil. 1998;12: 506–13. 9869254 10.1191/026921598672167702

[13] Laska AC, Mårtensson B, Kahan T, von Arbin M, Murray V. Recognition of depression in aphasic stroke patients. Cerebrovasc Dis. 2007;24: 74–9. 17519547 10.1159/000103119

[14] Cobley CS, Thomas SA, Lincoln NB, Walker MF. The assessment of low mood in stroke patients with aphasia: reliability and validity of the 10-item Hospital version of the Stroke Aphasic Depression Questionnaire (SADQH-10). Clin Rehabil. 2012;26: 372–81. 10.1177/0269215511422388 22023890

[15] Kauhanen ML, Korpelainen JT, Hiltunen P, Määttä R, Mononen H, Brusin E, et al Aphasia, depression, and non-verbal cognitive impairment in ischaemic stroke. Cerebrovasc Dis. 2000;10: 455–461. 11070376 10.1159/000016107

[16] Williams LS, Brizendine EJ, Plue L, Bakas T, Tu W, Hendrie H, et al Performance of the PHQ-9 as a screening tool for depression after stroke. Stroke. 2005;36: 635–8. 15677576 10.1161/01.STR.0000155688.18207.33

[17] de Man-van Ginkel JM, Hafsteindsdóttir T, Lindeman E, Burger H, Grobbee D, Schuurmans M. An efficient way to detect poststroke depression by subsequent administration of a 9-Item and a 2-Item Patient Health Questionnaire. Stroke. 2012;43: 854–6. 10.1161/STROKEAHA.111.640276 22156689

[18] Sulter G, Steen C, De Keyser J. Use of the Barthel index and modified Rankin scale in acute stroke trials. Stroke. 1999;30: 1538–1541. 10436097 10.1161/01.str.30.8.1538

[19] Sibon I, Lassalle-Lagadec S, Renou P, Swendsen J. Evolution of depression symptoms following stroke: a prospective study using computerized ambulatory monitoring. Cerebrovasc Dis. 2012;33: 280–5. 10.1159/000334663 22285959

[20] da Rocha e Silva CE, Alves Brasil MA, Matos do Nascimento E, de Braganca Pereira B, André C. Is poststroke depression a major depression? Cerebrovasc Dis. 2013;35: 385–91. 10.1159/000348852 23635428

[21] Gillen R, Tennen H, McKee TE, Gernert-Dott P, Affleck G. Depressive symptoms and history of depression predict rehabilitation efficiency in stroke patients. Arch Phys Med Rehabil. 2001;82: 1645–49. 11733876 10.1053/apmr.2001.26249

[22] NIH: National Institute of Mental Health Statistics. Major Depressive Disorder Among Adults. Available: http://www.nimh.nih.gov/statistics/1mdd_adult.shtml. Accessed 4 April 2014.

